# Reporting of patient and public involvement and engagement (PPIE) in clinical trials published in nursing science journals: a descriptive study

**DOI:** 10.1186/s40900-021-00331-9

**Published:** 2021-12-14

**Authors:** Richard Gray, Catherine Brasier, Tessa-May Zirnsak, Ashley H. Ng

**Affiliations:** 1 The School of Nursing and Midwifery, La Trobe University, Melbourne, VIC 3086 Australia; 2 Department of Social Work and Social Policy, La Trobe University, Melbourne, VIC 3086 Australia; 3 Department of Dietetics, Human Nutrition and Sport, La Trobe University, Melbourne, VIC 3086 Australia

**Keywords:** Patient and public involvement and engagement, PPIE, Clinical trials, Nursing

## Abstract

**Background:**

Patient and Public Involvement and Engagement (PPIE) in research positively affects the relevance, quality, and impact of research. Around 11% of studies published in leading medical journals demonstrate PPIE. The extent of PPIE in nursing research has not been previously studied.

**Methods:**

A descriptive study of PPIE in clinical trials published in general nursing science journals between 1st January and 31st August 2021. Data were extracted from included studies against the five items of the Guidance for Reporting Involvement of Patients and the Public (GRIPP2) short form reporting checklist.

**Results:**

We searched 27 journals and identified 89 randomised controlled clinical trials. There was no statement or evidence of PPIE in any of the included trials.

**Conclusion:**

Nurse researchers need to ensure that they purposefully involve patients in their research and report this in papers describing study findings.

**Supplementary Information:**

The online version contains supplementary material available at 10.1186/s40900-021-00331-9.

## Plain English summary

Patient involvement in research may improve the quality and relevance of the work. The British Medical Journal—one of the top medical research journals in the world—has developed a patient partnership plan. Part of this plan requires papers sent to the journal for publication to include a statement about how patients were involved in the research. If patients were not involved in the research, this should be stated in the paper. Most papers published in the British Medical Journal now include a statement about patient involvement. We wanted to check if nurses were reporting patient involvement in the research they do. We read 89 randomised controlled trials (RCTs) published in 27 general nursing journals. We focused on RCTs because they are widely considered the gold standard for evaluating interventions and therefore are most likely to impact on patient care. Unlike the British Medical Journal, there was no evidence that nursing journals required authors to say how patients were engaged or involved in the research they published. There was no evidence of patient involvement in any of the nursing research articles that we looked at. The implication of our work is that nurse researchers need to be developing ways for effective engagement of patients in all aspects of their research and explaining how they did this in the papers they publish in nursing journals.

**Supplementary Information:**

The online version contains supplementary material available at 10.1186/s40900-021-00331-9.

## Introduction

Patient and public involvement and engagement (PPIE) in research can be defined as research carried out “with” or “by” members of the public rather than “to”, “about” or “for” them (National Institute for Health Research, n.d. [4]). There is a consensus that PPIE in research improves the relevance, quality, and impact of the work [8, 12]. In PPIE, consumers are active partners in all aspects of research, from generating the research question, writing the grant application, developing the study protocol, collecting data, analysing and interpreting results and co-authoring publications [12].

Major research funding bodies are, to varying degrees, encouraging of PPIE. In the United Kingdom, for example, the National Institute for Health Research (NIHR)—the major funding body for applied health research—has taken a notably forthright position, requiring researchers applying for funding to “describe how they have involved the public in the design and planning of their study as well as their plans for further involvement throughout the research, including plans for evaluating impact” (National Institute for Health Research, n.d. [4]). In Australia, the National Health and Medical Research Council encourages researchers to involve patients in all stages of the research process (NHMRC, 2016).

While researchers seem to be generally positive about involving patients in research, they struggle to achieve this in practice [1]. For example, authors of a qualitative exploration of 36 researchers’ experiences of PPIE found that participants expressed a combination of ambivalence, cynicism and enthusiasm about PPIE [3]. Further, [3] report that participants described PPIE as both rewarding and burdensome, requiring practical and social support. Bowers et al. [2] argue that PPIE in research is time-consuming and more challenging for patients, the public and researchers than is often argued.

From a search using MEDLINE, we identified four studies that have examined the extent to which PPIE has been integrated into health research [5–8]. Owyang et al. [6] report a systematic review examining the prevalence and quality of PPIE in RCTs in orthopaedics. The authors reviewed 475 studies and identified two trials where there was some evidence of purposeful PPIE. In one, PPIE was used to inform the research question, choice of outcome and the dissemination of findings. In the second trial, PPIE informed the study design.

The *British Medical Journal* (*BMJ*) has been influential in promoting PPIE through its Patient Partnership Strategy. In 2014 the *BMJ* implemented a policy requiring authors to make a PPIE declaration in the methods section of submitted manuscripts. Authors that had not involved patients were asked to state this in the paper explicitly. This policy aimed to increase PPIE in research by shifting cultural expectations among clinical researchers. Price et al. [9] report the frequency of PPIE in research published in the *BMJ* before and after implementing the consumer involvement policy. Of 152 papers—reporting any study design—published in the year following the implementation of the policy, there was an increase from less than 1–11% of authors reporting PPIE, an important improvement but suggesting that consumer involvement in research is still infrequent.

Staniszewska et al. [11] discussed the development of the GRIPP2 (Guidance for Reporting Involvement of Patients and the Public version 2) checklist for reporting patient and public involvement in research. The authors used the EQUATOR method for developing reporting guidelines: a three-round Delphi study and consensus statement. GRIPP2 identified key concepts that authors should report to describe PPIE in their research. The short-form version of the guideline addresses five topics: (1) the aim of PPIE in the study, (2) methods used for PPIE, (3) results of PPIE in the study, (4) extent to which PPIE influenced the study overall, (5) reflections. Staniszewska et al. [11] suggest that journal editors can use the GRIPP2 checklist to set reporting expectations for submitted manuscripts.

The extent to which PPIE is demonstrated in nursing research has not been previously examined. Given that nursing is a patient focused discipline, PPIE would seem to be particularly important when designing trials evaluating new approaches to providing nursing care. We undertook a descriptive study to show to what extent nurses involve and engage patients in nursing randomised controlled clinical trials.

## Methods

We conducted a comprehensive descriptive study of PPIE in RCTs published in general nursing science journals listed in the 2020 Journal Citation Report. We identified key general nursing journals, located each of the publications published within these journals and then systematically screened each of these articles for reporting of PPIE.

### Ethical considerations

Ethical approval for this study was not required because data were extracted from publicly available sources or published research.

### Lived experience academic involvement

The study was instigated by RG, who does not identify as a lived experience researcher. AN, TZ and CB are employed as teaching and research academics and identify as having health related conditions. AN, TZ and CB have experience as experts and/or advocates in their diagnosed health conditions, and subsequently are lived experience researchers. All four research team members are committed to physical health patient, mental health consumer and stakeholder engagement and were involved across all stages of this project and held decision making roles.

### Pre-registration of the study methodology

The methodology for this study was registered with the Open Science Framework. The registration entry can be accessed via this link: https://osf.io/x2bqv.

#### Amendments to the methodology following study registration

Based on reviewer feedback we amended our search to include a broader list of general nursing journals than initially planned. We made amendments to the way in which we coded the country where the corresponding author was based, and funding information. Additionally, we extracted information from included manuscripts about authors who identified as lived experience researchers. Finally, the websites of funding agencies were checked to determine if PPIE was a requirement of funding.

### Journal selection process

General nursing journals were selected by reviewing the author guidelines of all nursing science journals that are listed in the 2020 Clarivate Journal Citation Report. General nursing journals were defined as those that published research manuscripts across multiple fields of practice. Journals that had a discreet clinical focus area (e.g., mental health, midwifery) were not included in the study.

### Identifying the articles from the selected journals

All peer reviewed papers published in included general nursing science journals were identified using the “sources” function in SCOPUS (https://www.elsevier.com/en-gb/solutions/scopus), entering the journal title, and accessing the “SCOPUS content coverage” tab. We then restricted documents to those published to date in 2021 (1st January through 31st August 2021).

### Inclusion criteria for the articles

Published manuscripts reporting the primary analysis of a RCT.

### Screening the identified articles

All papers published in included journals were downloaded from the SCOPUS database, exported into Covidence and screened by title, abstract and full text [RG, AN, CB] according to the inclusion criteria. Any conflicts were resolved by discussion or by a third author.

### Data extraction

Included papers were read in full—including footnotes and acknowledgements—by two researchers [from a team of four CB, RG, AN, TZ] who independently extracted the following information on to a custom spread sheet: citation, summary of the trial, the clinical focus of the trial (coded: 1. Medical, 2. Surgical, 3. Obstetric, 4. Mental health, 5. Children, 6. Emergency department), country where fieldwork was conducted (subsequently recoded into the following United National geo-scheme zones: 1. Asia, 2. The Americas, 3. Europe, 4. Oceania), if any authors reported as being a lived experience researcher (meaning they were employed in an identified research role), a summary of PPIE reported in the manuscript, if participants were thanked in the acknowledgement section of the manuscript, source(s) of funding (coded: 1. own account, 2. University, 3. not reported, 4. external grant). Finally, information against the five GRIPP2 short-form items was extracted.

#### PPIE requirement of funding bodies

We checked the website of funding organisations identified in included manuscripts as providing external funding to trials to determine if PPIE was a recommendation or requirement of funding applications. This was extracted onto the custom spreadsheet described above.

#### Journal author guidelines

Two researchers [RG, TZ] read and extracted a link to the author guidelines of included journals to identify any requirements to report PPIE in submitted manuscripts. We also checked if authors were instructed to thank patients involved in studies in the acknowledgement section of the manuscript. This was extracted onto a separate custom spreadsheet.

## Results

### Included journals

We identified 27 general nursing journals (Additional file 1: list of included journals) that were included in the study.

### Journal author guidelines

The author guidelines of the 27 included journals neither implicitly nor explicitly required authors to report how patients were involved in the research submitted to the journal. All journals either required or encouraged that authors follow relevant reporting guidelines listed on the EQUATOR network but did not mention GRIPP or GRIPP2.

The author guidelines of the included journals did not state that it was a requirement that authors thank participants for their contribution to the research.

### Included studies

**Fig. 1:**
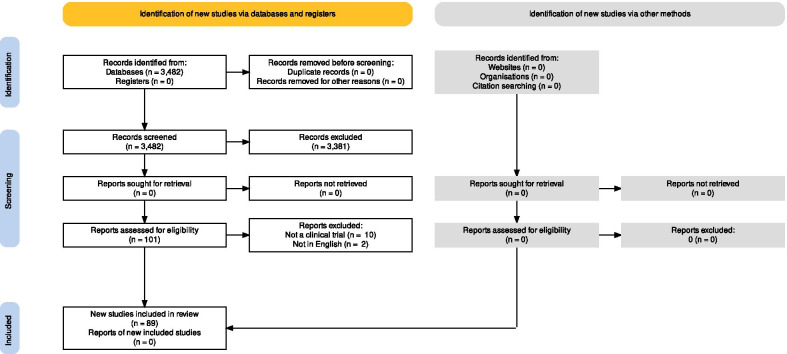
Flow of papers through the study

Figure 1 shows the flow of papers through the study. As of the end of August 2021, the 27 included journals had published 3482 documents that were listed on the SCOPUS website. Fourteen journals published at least one RCT. In total we identified 89 papers reporting the results of a RCT. A complete list of included studies is available in Additional file 2:  list of included trials.

Additional file 3:  Data extraction shows the information extracted from included studies. Table 1 summarises these data. Over two thirds of the corresponding authors were based in Asia (n = 64, 72%) most studies were focused on patients with a medical condition (n = 62, 70%), half (n = 46, 52%) were funded by an external grant.

**Table 1:** Summary of included trials

	Number (%)
*Clinical population*	
Medical	62 (70%)
Surgical	6 (7%)
Obstetric	7 (8%)
Mental Health	11 (12%)
Children	1 (1%)
Emergency department	2 (2%)
*Region where fieldwork was conducted*	
Asia	64 (72%)
Americas	8 (9%)
Europe	16 (18%)
Oceania	1 (1%)
*Funding*	
Own account	22 (25%)
University	7 (8%)
Not reported	14 (16%)
External grant	46 (52%)
Summary of PPIE in the manuscript	0 (0%)
Participants were thanked in the acknowledgement section of the manuscript	34 (38%)
At least one author on an included manuscript identified as a lived experience (patient) researcher	0 (0%)
*GRIPP 2—short form*	
Aim (the aim of PPI in the study)	0 (0%)
Methods (description of the methods used for PPI in the study)	0 (0%)
Study results (results of PPI in the study)	0 (0%)
Discussion and conclusions (the extent to which PPIE influenced the study overall)	0 (0%)
Reflections and critical perspective	0 (0%)

### Patient and public involvement and engagement

None of the researchers listed as authors on included studies were identified as lived experience researchers (Table 1).

Authors of almost two thirds (n = 55, 62%) of the included studies did not thank participants for their contribution to the research in the acknowledgement section of the manuscript.

A summary of PPIE was not reported in any of the included manuscripts. There was no evidence of patient or public involvement in any of the included studies; consequently, we could not rate any of the GRIPP2 short-form items.

### PIE requirements of funding bodies

We searched the websites of funding bodies that had supported included trials. The National Institute for Health Research (NIHR) does require researchers to demonstrate PPIE as part of grant application. We did not locate evidence that PPIE was a requirement of any other funding body. Two funding websites were exclusively available in French, and we were not able to review these.

## Discussion

This study aimed to investigate the reporting of PPIE in clinical trials published in general journals in nursing. We found no evidence of PPIE in any of the 89 trials that we reviewed. Our finding is consistent with similar studies in other clinical disciplines [5–7]. For example, Owyang et al. [6] found evidence of patient and public involvement in only two of the 475 orthopaedic trials they included in their review. Notably, much higher rates of PPIE have been reported in the *British Medical Journal*, where 1 in 10 studies have some form of PPIE activity [8].

None of the 27 nursing science journals we included in this review required authors to report PPIE in the papers they publish. Again, authors of studies in other disciplines reported similar findings. For example, in their study of PPIE in orthodontic research, Patel et al. [7] reported that none of the four journals they included had guidance on PPIE. The exception is the *British Medical Journal* that actively promotes PPIE in research they publish. The *BMJ* introduced a policy in 2014 requiring authors submitting manuscripts to the journal to include a PPIE declaration in their methods section [8].

Trials published in nursing science journals were seemingly not supported by funding bodies that required PPIE as part of the grant application process. Our observation is somewhat consistent with other disciplines; for example, Patel et al. [7] reported in their study of PPIE that of nine funding agencies that they reviewed, only two (the National Institute for Health Research and Health Care Research Wales) required applicants to demonstrate PPIE as part of the application process.

The reporting of RCTs requires that authors follow CONSORT reporting guidelines [10]. We note that it is not a requirement of these guidelines to report PPIE. It may be that authors of trials included in this descriptive study did not report PPIE because it is not an item in the CONSORT guidelines. Those responsible for developing revised CONSORT guidelines might consider the reporting of PPIE an important part of full and transparent reporting of clinical trials in future revisions.

It is part of the *BMJ* patient partner strategy that authors should thank the patients that participated in the research in the acknowledgement section of their manuscript [8]. Expressing gratitude to people giving freely of their time in the advancement of knowledge seems to us important. It is the least that researchers can do, particularly as published research articles are often inaccessible to patients as they are either behind a paywall or jargon-heavy and challenging for the general public to understand. That most of the included nursing trials did not thank patients for their contribution is disappointing.

### Limitations

It may be that researchers are involving patients in their research and then not reporting this in the published manuscript. In their study of PPIE rates, Patel et al. [7] attempted to check—by emailing authors—if there was PPIE in the trial that had not been reported in the manuscript. Only 14% responded to the researcher’s request, suggesting that directly contacting authors may not be an effective way to check unreported PPIE [7]. Based on this observation, we concluded that there was little merit in contacting authors to check unreported PPIE. It may be that qualitative interviews with researchers may be a more productive approach to addressing this issue.

Identifying if authors of included studies were lived experience researchers was problematic. We assumed that it would be evident in the author affiliation if this were the case. However, the majority of the included journals did not require authors to report their job titles, and of the few that did, no authors reported holding a lived experience role. We also note that there is a culture in academia not to identify as lived experience academic. So even though authors may have an academic affiliation, they may still be a lived experience academic, but we would not know.

We note that it was difficult to determine if funding bodies required PPIE because of how information was organised on websites. It may be that PPIE was a requirement, but this was reported in parts of the funder’s website or in documents that we did not search. Potentially the the only way to check if PPIE is a requirement of funders is to contact them directly.

We only included trials published in 2021. It is possible that we would have observed more PPIE if we had included papers from preceding years. It is also possible that the COVID-19 pandemic may have negatively impacted the reporting of PPIE. That said, the fieldwork for most included trials was completed prior to the start of the COVID-19 pandemic, Further, the trend from other disciplines [3] would seem to suggest that over time we might expect growth in levels of PPIE reported in papers.

### Generalisability

The generalisability of our observation is limited by our focus on nursing journals and our decision to only focus on papers reporting clinical trials. We included general nursing journals that publish around 5,000 papers a year and are listed in the Journal Citation Report. This represents a small fraction of the nursing journals that are published each year. It may be that there are higher levels of PPIE reported in papers in more specialist nursing journals. For example, we are aware that the *Journal of Psychiatric and Mental Health Nursing* has a specific section on lived experience narratives. We focused on clinical trials because this design is likely to have a direct impact on patient care, making PPIE, arguably, more important. It may be that PPIE is more prevalent in observational and qualitative research and systematic reviews.

Nurses may publish their research in non-nursing journals because they may perceive them as more prestigious or subject relevant. It is plausible that these studies are more likely to report effective PPIE engagement than those in nursing journals. Consequently, we cannot generalise our observations to all trials by nurses.

### Lived experience (patient) academic perspectives (NG, TZ and CB) on the study findings

The original premise and intent of PPIE in research is to ensure that the resulting work benefits the community it serves. Partnerships with various stakeholders in research are critical to co-design and develop solutions that are more likely to be implemented and used within practice. Patients, consumers, and the general public need to be seen as stakeholder as important as clinicians and policymakers who are often included in research design and implementation.

Results from this study highlight that while there is a slow culture change to acknowledge patients and the public as important equals in research, there is still much to be done across journals and research institutions alike. It is not surprising that researchers often focus on the barriers when it comes to PPIE in research, which is perhaps the next knot to untangle in embracing PPIE.

### Lived experience academic (AN, TZ and CB) experiences of involving patients and the public in research

Including PPIE in research provides a different perspective from researchers and clinicians when it comes to tackling issues in healthcare. Patients and the public are privy to the challenges they face when navigating the healthcare system that may be unbeknownst or considered by researchers or clinicians. However, it can be challenging to find patients and the public who can speak freely amongst the research team, particularly when hierarchical systems are in place. Additionally, while PPIE can be embedded in research grants, it can sometimes be done in a tokenistic manner, diminishing the value of true PPIE and increasing cynicism in patients to be involved with future projects.

## Conclusions

Despite agreement about the value of PPIE in improving the relevance and impact of research, we found no evidence that nurses had purposefully engaged people with lived experience in clinical trials. Further, authors frequently did not thank patients for their contribution to the research in the acknowledgement section of their manuscripts. Researchers, universities, clinical services, funding agencies and journal editors need to engage in strategies to promote effective and purposeful PPIE across all aspects of the research process.

## Supplementary Information

**Additional file 1.** List of included journals.

**Additional file 2.** List of included trials.

**Additional file 3.** Data extraction table.

## Data Availability

A list of included studies and the data extraction table are available as supplementary documents.

## Abbreviations

PPIE: Patient and public involvement and engagement
BMJ: British Medical Journal
GRIPP2: Guidance for reporting involvement of patients and the public
NIHR: National Institute for Health Research
NHMRC: National Health and Medical Research Council
