# Supplementary material for: Reporting of patient and public involvement and engagement (PPIE) in clinical trials published in nursing science journals: a descriptive study
Source: Res Involv Engagem. 2021 Dec 14;7:88. doi: 10.1186/s40900-021-00331-9 (PMC8669663; doi:10.1186/s40900-021-00331-9)
Supplement: Supplementary file 2 — Additional file 2. List of included trials. [file 40900_2021_331_MOESM2_ESM.docx]

**Supplementary document 3, patient and public involvement and engagement in reports of results of clinical trials using GRIPP 2-SF**

|  |  | | | | | | | GRIPP 2 – short form items | | | | |
| --- | --- | --- | --- | --- | --- | --- | --- | --- | --- | --- | --- | --- |
| Study number | Citation | Summary of trial | Country where corresponding authors was based | Summary of PPIE in the manuscript | Participants were thanked in the acknowledgement section of the manuscript | Funding information | At least one of the authors Identifies as having lived experience (as a patient) | 1. Aim (the aim of PPIE in the study) | 2. Methods (description of the methods used for PPIE in the study) | 3. Study results (results of PPIE in the study) | 4. Discussion and conclusions (the extent to which PPIE influenced the study overall) | 5. Reflections/ critical perspective |
| 1 | Abanes et al (2021) | RCT into impact of acupuncture combined with CBT in military service persons with insomnia. Participants randomised to the acupuncture group reported improvements to insomnia severity | Japan | None | No | Own account | Job titles of authors not reported | Not reported | Not reported | Not reported | Not Reported | Not reported |
| 2 | Akgün Kostak et al (2021) | Prospective RCT examining the role of finger puppet play on patient stress in patients 5-10 years prior to surgery. Results indicate that patients in the intervention group and their parents experienced less stress pre and post op. | Turkey | None | Yes | Own account | Job titles of authors not reported | Not reported | Not reported | Not reported | Not reported | Not reported |
| 3 | Akhlaghi et al (2021) | Two-8arm RCT evaluating the efficacy of the Neuman Systems model to reduce stress in patients undergoing a coronary artery bypass graft. There was a statistically significant reduction in stress in the intervention group at the conclusion of the trial | Iran | None | Yes | University funded master’s scholarship | No | Not reported | Not reported | Not reported | Not reported | Not reported |
| 4 | Akin et al (2021) | Two arm RCT examining if dance during labour increased patient comfort and lowered patient trauma following birth. Result suggest that dance did achieve these aims. | Turkey | None | No | Own account | Not stated | Not reported | Not reported | Not reported | Not reported | Not reported |
| 5 | Al-Jubouri et al(2021) | This three-arm experimental comparative study examined the impact of listening to music or the Koran in reducing stress prior to chemotherapy. Results indicated that Koran or music were equally effective in reducing stress. | Iraq | None | No | Not reported | Job titles of authors not reported | Not reported | Not reported | Not reported | Not reported | Not reported |
| 6 | Arazi et al (2021) | RCT of people on haemodialysis with respiratory exercise and 6 minute walk each day for 2 months as intervention. Results indicate the intervention was effective. | Iran | None | Yes | Not reported | No | Not reported | Not reported | Not reported | Not reported | Not reported |
| 7 | Arjunan, & Trichur (2021) | This two arm RCT sought to evaluate if a nurse-led cardiac rehabilitation program improved quality of life, Results indicated that it did. | India | None | No | Not reported | No | Not reported | Not reported | Not reported | Not reported | Not reported |
| 8 | Barbour et al (2021) | Two arm RCT examining if a topical analgesic spray prevented pain in needlestick procedures. Both patients and healthcare providers reported a decrease in pain as compared to the placebo group | USA | None | No | The Gebauer company | Job titles of authors not reported | Not reported | Not reported | Not reported | Not reported | Not reported |
| 9 | Basak et al (2021) | 91 patients prescribed intramuscular antibiotic medication were randomised to virtual reality, distraction cards or control conditions. Pain levels were lower in both virtual reality and distraction card conditions compared to control. | Turkey | None | Yes | Not reported | No | Not reported | Not reported | Not reported | Not reported | Not reported |
| 10 | Bielderman et al (2021) | Two arm RCT examining the short and long-term effects of the TENSE training program on experienced stress, work contentment, and stress reactions at work in nursing staff working in dementia special care units. Findings suggest the intervention was not effective | The Netherlands | None | No | Vitalis WoonZorg Groep, Eindhoven, The Netherlands. | Job titles of authors not reported | Not reported | Not reported | Not reported | Not reported | Not reported |
| 11 | Bozdogan Yesilot et al (2021) | Two arm RCT examining if virtual reality headsets were an effective way to decrease moderate-level anxiety in mothers whose children were being circumcised. Quantitive outcomes suggest virtual reality is effective. | Turkey | Not reported | No | Own account | No | Not reported | Not reported | Not reported | Not reported | Not reported |
| 12 | Bunsanong and Chaimongkol (2021) | RCT of a self-management intervention for “middle-aged” women with knee osteoarthritis. Self-management resulted in better knee functional status compared to TAU | Thailand | None | Yes | University funding | Job titles of authors not reported | Not reported | Not reported | Not reported | Not reported | Not reported |
| 13 | Calvo et al (2021) | Two arm RCT assessing the impact of a nursing intervention on therapeutic adherence in elderly patients after myocardial infarction. Findings indicate the intervention group were more likely to adhere to their treatment regime. | Spain | None | No | Health Research and Innovation Strategic Plan intensification grant, Generalitat de Catalunya. | Job titles of authors not reported | Not reported | Not reported | Not reported | Not reported | Not reported |
| 14 | Çankaya, & Şimşek (2021) | 120 pregnant women were assigned 1:1 to antenatal education program or TAU. Participants in the education program reported less fear around birthing than the control group | Turkey | None | Yes | Own account | No | Not reported | Not reported | Not reported | Not reported | Not reported |
| 15 | Cayir et al (2021) | RCT examining the impact of taking a one-minute pause after the death of a patient on healthcare providers. This pause did contribute to a lowering of heart rate, but had no impact on blood pressure | USA | None | No | Private grant | Job titles of authors not reported | Not reported | Not reported | Not reported | Not reported | Not reported |
| 16 | Chan et al (2021) | Two arm RCT examining the effect of virtual reality-based documents on knowledge and attitude towards chemotherapy administration in nursing students. Findings suggest VR can address the shortcomings of the traditional learning resources. | Taiwan | None | No | Own account | Job titles of authors not reported | Not reported | Not reported | Not reported | Not reported | Not reported |
| 17 | Chen et al (2021) | Two arm RCT investigating efficacy of a home-based intervention program for stroke survivors with lower limb spasticity. While patients in both cohorts improved, patients in the intervention group improved more substantially | China | None | No | Wenzhou Science and Technology Bureau | Job titles of authors not reported | Not reported | Not reported | Not reported | Not reported | Not reported |
| 18 | Chew et al (2021) | Push-pull-hold program compared to TAU in 144 heart failure patients. Self-care improved in the experimental compared to the TAU group. | Singapore | None | No | PhD scholarship | Job titles of authors not reported | Not reported | Not reported | Not reported | Not reported | Not reported |
| 19 | Choi & Kim (2021) | This RCT examined if application of uncoated paper to skin reduced incidence of pressure injuries. The intervention did lower the moisture level of the skin in the intervention group, but there was no significant difference between control and intervention groups | Korea | None | No | Own account | No | Not reported | Not reported | Not reported | Not reported | Not reported |
| 20 | Chung et al (2021) | This RCT compared adventure-based training as opposed to leisure activities in children aged 12-16. Students in the intervention group reported higher resilience and fewer depressive symptoms at follow up | Hong Kong | None | Yes | Knowledge Exchange Funding Exercise 2018/19, Impact Project Scheme, The University of Hong Kong | Job titles of authors not reported | Not reported | Not reported | Not reported | Not reported | Not reported |
| 21 | Deng et al (2021) | Patients under mechanical ventilation received low-dose remifentanil, high-dose remifentanil or placebo prior to each tracheal suctioning in a random order. Both low dose and high dose groups reported lower agitation than the placebo group. | China | None | No | Sichuan Science and Technology Program | No | Not reported | Not reported | Not reported | Not reported | Not reported |
| 22 | de Pinho et al (2021) | Metacognitive training for 56 people with schizophrenia. Results indicate the intervention was effective at reducing psychotic symptoms compared with TAU. | Portugal | None | No | The Portuguese Society of Mental Health Nursing; CINTESIS R&D Unit. | Job titles of authors not reported | Not reported | Not reported | Not reported | Not reported | Not reported |
| 23 | Dincer and Yildrim (2021) | 84 patients attending the emergency department were randomised to 24receive vibration stimulation or treatment as usual to reduce pain associated with injection. | Turkey | None | Yes | Own account | No | Not reported | Not reported | Not reported | Not reported | Not reported |
| 24 | Diniz et al (2021) | RCT examining telephone consultations in anticipation of bowel care for colonoscopy. Intervention group had demonstrated better bowel care at time of colonoscopy | Brazil | None | No | Coordenação de Aperfeiçoamento de Pessoal de Nível Superior e Conselho Federal de Enfermagem | No | Not reported | Not reported | Not reported | Not reported | Not reported |
| 25 | Doğan, U., Ovayolu (2021) | Two arm RCT investigating the effect of nurse-administered training on treatment adherence of coronary artery patients. | Turkey | None | Yes | Own account | Job titles of authors not reported | Not reported | Not reported | Not reported | Not reported | Not reported |
| 26 | Durmuş İskender, & Çalışkan (2021) | Three arm RCT examining the impact of acupressure on constipation in people with total knee arthroplasty. Acupressure and abdominal massage seem to relieve constipation in this group. | Turkey | None | Yes | Own account | No | Not reported | Not reported | Not reported | Not reported | Not reported |
| 27 | Eslami et al (2021) | Two arm RCT in overweight or obese pregnant women to examine if a lifestyle training package increased nutrition quality and physical activity. Findings suggest that the intervention did increase these two outcomes | Iran | None | Yes | Tabriz University of Medical Sciences | Job titles of authors not reported | Not reported | Not reported | Not reported | Not reported | Not reported |
| 28 | Evans et al (2021) | Two arm RCT evaluating the impact of a short-term integrated palliative and supportive care intervention for older people living with chronic noncancer conditions and frailty on clinical and economic outcomes and perceptions of care. Findings indicate that the intervention was effective. | England | None | No | National Institute for Health Research (NIHR) Research for Patient Benefit Programme; Health Education England/ NIHR Clinical Lectureship and Senior Clinical Lectureship; NIHR Career Development Fellowship; NIHR Clinician Scientist Fellowship; NIHR Applied Research Collaboration; NHS Foundation Trust. | Job titles of authors not reported | Not reported | Not reported | Not reported | Not reported | Not reported |
| 29 | Franco-Antonio et al (2021) | Two arm RCT examining a brief motivational intervention for mothers engaging in breastfeeding. Results indicate the intervention increased mother’s confidence toward breastfeeding. | Spain | None | No | Johnson and Johnson; International Confederation of Midwives Research Award 2018; Programa Operativo FEDER Extremadura (2014‐2020) | Job titles of authors not reported | Not reported | Not reported | Not reported | Not reported | Not reported |
| 30 | Hajialibeigloo et al (2021) | Two-arm RCT examining a self-administration program versus usual care for adherence to medication. Results suggest the intervention is effective | Iran | None | Yes | Research Deputy of Mashhad University of Medical Sciences, Mashhad, Iran | Job titles of authors not reported | Not reported | Not reported | Not reported | Not reported | Not reported |
| 31 | Hassan et al (2021) | RCT testing the Trendelenburg position with comparator in obese critically ill patients. The experimental position led to improved respiratory parameters in 110 participants | Egypt | None | Yes | Not reported | No | Not reported | Not reported | Not reported | Not reported | Not reported |
| 32 | Ho et al (2021) | Two arm RCT evaluating a simulation-based education programme on critical care nurses’ knowledge, and clinical performance in providing delirium care. Findings indicate the intervention is effective. | Taiwan | None | No | Taipei Medical University-Wan Fang Hospital; University of Wollongong | Job titles of authors not reported | Not reported | Not reported | Not reported | Not reported | Not reported |
| 33 | Hong et al (2021) | Randomized waitlist-controlled clinical trial examining health information technology on self-management technology. Results indicate efficacy of the intervention and increased quality of life. | Taiwan | None | Yes | Tri-Service General Hospital R.O.C. | Job titles of authors not reported | Not reported | Not reported | Not reported | Not reported | Not reported |
| 34 | Huang et al (2021) | Two arm RCT testing a mobile app for graduate nurses on the use and care of central venous catheters. There was some evidence that the intervention was effective. | Taiwan | None | Yes | Hualien Tzu Chi Hospital | Job titles of authors not reported | Not reported | Not reported | Not reported | Not reported | Not reported |
| 35 | Hung et al (2021) | RCT examining the use of auricular acupressure on gendered experiences of poor sleep quality. Results indicate that the intervention was effective for the intervention group, with men experiencing increased efficacy of the intervention | Taiwan | None | No | Ministry of Science and Technology, Taiwan, ROC | No | Not reported | Not reported | Not reported | Not reported | Not reported |
| 36 | Jiang et al (2021) | Three arm RCT evaluating the effect of a nurse-led, home-based self-management psychosocial education intervention. Findings suggested that the interventions were effective for patients with heart failure in Singapore. | Singapore | None | No | Health Services Research Grant from National Medical Research Council (NMRC). | Job titles of authors not reported | Not reported | Not reported | Not reported | Not reported | Not reported |
| 37 | Kaplan Serin, & Citlik Saritas (2021) | RCT evaluating the effect of TMT-based training and follow-up on improving walking exercise behaviour  and metabolic control in patients with type 2 diabetes. There was a significant increase in steps among the intervention groups. | Turkey | None | No | Own account | Job titles of authors not reported | Not reported | Not reported | Not reported | Not reported | Not reported |
| 38 | Kes, & Polat (2021) | RCT evaluating telephone monitoring on medication adherence and blood pressure (BP) control in primary hypertension. Medication adherence was significantly higher in the intervention group. | Turkey | None | No | Own account | No | Not reported | Not reported | Not reported | Not reported | Not reported |
| 39 | Kim et al (2021) | RCT using a pre-post design to evaluate a smartphone application for management of diabetes. The intervention group reported decreased sugar, fat, and carbohydrate intake. | South Korea | None | No | Own account | Job titles of authors not reported | Not reported | Not reported | Not reported | Not reported | Not reported |
| 40 | Köse, & Yıldız (2021) | Two arm RCT evaluating a motivational program for obese adolescents. After 6 months, the body mass index values had decreased in the intervention group compared with baseline, with no decrease in the control group. | Turkey | None | No | Scientific Research Projects Coordination Unit of Istanbul University | Job titles of authors not reported | Not reported | Not reported | Not reported | Not reported | Not reported |
| 41 | Koundal et al (2021) | Two arm RCT testing the effectiveness of a nurse-led dietary diabetes insipidus (DI) bundle on the severity of postoperative fluid imbalance in pituitary region tumours. Findings indicate the intervention was effective | India | None | No | Own account | Job titles of authors not reported | Not reported | Not reported | Not reported | Not reported | Not reported |
| 42 | Lee et al (2021) | Two arm RCT evaluating the effect of a patient-centred self-management programme on mental health and self-management of patients with hypertensive nephropathy. Findings indicate that the intervention was effective. | Taiwan | None | No | Ministry of Science and Technology, Taiwan | Job titles of authors not reported | Not reported | Not reported | Not reported | Not reported | Not reported |
| 43 | Lee & Cha (2021) | Two arm RCT examining a virtual self-healing intervention as compared to audio-guided meditation. The effect of the intervention was moderate. | South Korea | None | No | Basic Science Research Program through the National Research Foundation of Korea (NRF) funded by the Ministry of Education | No | Not reported | Not reported | Not reported | Not reported | Not reported |
| 44 | Lescop et al (2021) | Non-inferiority trial testing the Buzzy device with lidocaine path in children undergoing a needle-related procedure. A total of 219 children were randomised. Pain levels were lower in the comparator compared to the Buzzy device group. | France | None | Yes | French Ministry of Health | Job titles of authors not reported | Not reported | Not reported | Not reported | Not reported | Not reported |
| 45 | Liao et al (2021a) | Two arm RCT evaluating the effectiveness of green tea mouthwash for improving the oral health status in oral cancer patients. Findings indicate the intervention is effective. | Taiwan | None | Yes | Ditmanson Medical Foundation, Chia-Yi Christian Hospital | Job titles of authors not reported | Not reported | Not reported | Not reported | Not reported | Not reported |
| 46 | Liao et al (2021b) | Three arm RCT examining the effects of mothers’ voice and white noise on sleep-wake patterns, salivary cortisol levels, weight gain, heart rate, and oxygen saturation of premature infants in a neonatal intensive care unit. Findings indicated the intervention may be useful for weight gain only. | China | None | Yes | Young Backbone Personnel Training Project, Health Department of Fujian Province in China | Job titles of authors not reported | Not reported | Not reported | Not reported | Not reported | Not reported |
| 47 | Liebergall-Wischnitzer et al. (2021) | 64 women following elective caesarean section randomised to receive Paula method exercises or treatment as usual. The exercises were effective at promoting the resumption of gastrointestinal activity | Israel | None | No | Not reported | Job titles of authors not reported | Not reported | Not reported | Not reported | Not reported | Not reported |
| 48 | Lin et al (2021) | Three arm RCT evaluating the Care & Organize Our Lifestyle and a self-regulation theory-based mHealth programmes on improving disease knowledge in youth with congenital heart disease. Findings indicate that the intervention was not effective. | Taiwan | None | Yes, but referred to as “study subjects” | Ministry of Science and Technology in Taiwan | Job titles of authors not reported | Not reported | Not reported | Not reported | Not reported | Not reported |
| 49 | Liu et al (2021) | Two arm RCT assessing effects of exercise as an intervention in cognitive function in women with methamphetamine dependence. The intervention led to some improvements on some cognitive processes. | China | None | Yes | Science &Technology Development Fund of Tianjin Education Commission for Higher Education | No | Not reported | Not reported | Not reported | Not reported | Not reported |
| 50 | Lyu et al (2021) | Two arm RCT evaluating the effects of a web-based transitional program on the glycaemic control and quality of life of Chinese patients with type 2 diabetes and explore the roles of self-efficacy and treatment adherence. Findings indicate that the intervention was effective. | China | None | Yes | Own account | Job titles of authors not reported | Not reported | Not reported | Not reported | Not reported | Not reported |
| 51 | Manzato et al (2021) | Two arm RCT evaluating the effect of telephone follow-up on health-related quality of life and anxiety and depression symptoms in individuals starting warfarin therapy. Findings suggest the intervention is effective. | Brazil | None | No | Coordination for the Improvement of Higher Education Personnel; National Council for Scientific and Technological Development | Job titles of authors not reported | Not reported | Not reported | Not reported | Not reported | Not reported |
| 52 | Mardani et al (2021) | Parallel RCT on impact of exercise on quality of life in prostate cancer survivors. In the intervention group, statistically significant improvements in physical, emotional, social and sexual functions, reduced insomnia and greater regularity of bowel movements were reported | Norway | None | Yes | University funded Masters account; Nord University, Norway (Publication costs only) | Job titles of authors not reported | Not reported | Not reported | Not reported | Not reported | Not reported |
| 53 | Milazi et al (2021) | Self-management of was more effective than treatment as usual in helping patients with end-stage kidney disease achieve phosphate control | Australia | None | Yes | Royal Brisbane and Women's Hospital Research Foundation, Health and Medical Research Unit, Queensland Health, Nursing and Midwifery Research Fellowship; The Queensland Nurses and Midwives’ Union. | Job titles of authors not reported | Not reported | Not reported | Not reported | Not reported | Not reported |
| 54 | Morales-Fernández et al. (2021) | RCT examining nurse facilitated intervention (healthy lifestyles, education on self-esteem, pain awareness, communication, and relaxation) compared to TAU in 279 patients with chronic malignant pain. | Spain | None | No | Government | Job titles of authors not reported | Not reported | Not reported | Not reported | Not reported | Not reported |
| 55 | Nesset et al (2021) | RCT comparing cognitive behaviour therapy (CBT) or mindfulness in 125 men that had perpetrated intimate partner violence. No difference against the primary outcome – anxiety and depression – was reported between the groups | Norway | None | No | Own account | Job titles of authors not reported | Not reported | Not reported | Not reported | Not reported | Not reported |
| 56 | Oh & Hwang (2021) | RCT examining an individualized educational intervention on the knowledge, attitudes, and self-management ability for outpatients with atrial fibrillation. The intervention group showed increased knowledge of stroke prevention | South Korea | None | No | Not reported | No | Not reported | Not reported | Not reported | Not reported | Not reported |
| 57 | Ok, & Kutlu (2021) | Two arm RCT examining the effect of motivational interviewing on adherence to treatment and quality of life in chronic haemodialysis patients. Increase in adherence to treatment was observed in the experimental group compared to the control group | Turkey | None | Yes | Istanbul University Scientific Research Projects. | No | Not reported | Not reported | Not reported | Not reported | Not reported |
| 58 | Østergaard et al (2021) | A RCT to test family nursing therapeutic conversations in 468 people with heart failure and their family. Social support improved in the intervention compared to the control. | Denmark | None | Yes | The Health Foundation; Danish Heart Foundation; Novo Nordisk Foundation. | No | Not reported | Not reported | Not reported | Not reported | Not reported |
| 59 | Pazarcikci & Efe (2021) | Two arm RCT examining the effect of a care programme based on the Comfort Theory on reducing parental anxiety during the perioperative process of paediatric day surgery. Results indicate that the intervention was effective. | Turkey | None | Yes | Scientific Research Projects Coordination Unit, Akdeniz University | Job titles of authors not reported | Not reported | Not reported | Not reported | Not reported | Not reported |
| 60 | Riera-Sampol et al (2021) | RCT investigating the effectiveness of nurse delivered motivational interviewing and exercise prescription compared with TAU in 263 with at least two cardiovascular risk factors | Spain | None | No | Fondo de Investigaciones Sanitarias of Instituto de Salud Carlos III. | Job titles of authors not reported | Not reported | Not reported | Not reported | Not reported | Not reported |
| 61 | Sahebkar et al (2021) | RCT testing different injection sites in patients attending the emergency department. Choosing an injection site based on body shape pattern was associated with lower levels of reported pain | Iran | None | Yes | Master’s degree project | No | Not reported | Not reported | Not reported | Not reported | Not reported |
| 62 | Samami et al (2021) | Two arm RCT investigating the effect of a supportive program on coping strategies and stress in women with breast cancer. The intervention group experienced a significantly higher increase on their problem-oriented coping strategies score. | Iran | None | No | Mazandaran University of Medical Sciences & Student Research Committee of Mazandaran University of Medical Sciences | Job titles of authors not reported | Not reported | Not reported | Not reported | Not reported | Not reported |
| 63 | Shariati et al (2021) | Two arm RCT identifying the effect of web-based communication between a nurse and a family member of a patient with COVID-19 on their perceived stress. The intervention group reported lower stress scores at the conclusion of the trial | Iran | None | Yes | Own account | No | Not reported | Not reported | Not reported | Not reported | Not reported |
| 64 | Shen et al (2021) | One-hundred patients with COPD randomised to active cycle breathing technique or TAU. Sputum production – the primary outcome – increased in the experimental compared to control intervention | China | None | No | Own account | Job titles of authors not reported | Not reported | Not reported | Not reported | Not reported | Not reported |
| 65 | Shin et al (2021) | Four arm RCT examining the effects of different pain relief during needle insertion into an implanted venous access chemoport. The three interventions showed evidence of some reduction of pain. | Republic of Korea | None | No | Not reported | Job titles of authors not reported | Not reported | Not reported | Not reported | Not reported | Not reported |
| 66 | Siebmanns et al (2021) | Two arm RCT examining cognitive behavioural therapy for insomnia in cardiovascular patients. Participants in the intervention group reported reduced insomnia that was maintained at 6-month follow up. | Sweden | None | No | School of Health and Welfare, Jönköping University, Jönköping, Sweden. | Job titles of authors not reported | Not reported | Not reported | Not reported | Not reported | Not reported |
| 67 | Silva et al (2021a) | Two arm RCT examining if educations for companions of people in labour improves the quality of support they can provide. Participants in the intervention group were more likely to provide support. | Brazil | None | No | Not reported | Job titles of authors not reported | Not reported | Not reported | Not reported | Not reported | Not reported |
| 68 | Silva et al (2021b) | Two arm RCT evaluating effectiveness of heparin solution compared to isotonic saline solution in preventing occlusion of the double lumen Hickman® catheter in patients undergoing hematopoietic stem cell transplantation. Results indicate that blocking with heparin solution is more effective than isotonic saline in preventing occlusion of the Hickman® catheter. | Brazil | None | No | Master’s degree project | Job titles of authors not reported | Not reported | Not reported | Not reported | Not reported | Not reported |
| 69 | Simonetti et al (2021) | Training in self-monitoring of blood pressure compared with TAU in people with hypertension improved patient adherence to treatment | Italy | None | No | Own account | No | Not reported | Not reported | Not reported | Not reported | Not reported |
| 70 | Simón-López et al (2021) | Three arm randomised cross over trial comparing heat, pressure, or heat and pressure, against control in 62 healthy volunteers. The primary outcome was successful venous catherization at the first attempt. High pressure was the most effective intervention | Spain | None | No | Own account | Job titles of authors not reported | Not reported | Not reported | Not reported | Not reported | Not reported |
| 71 | Sümen and Öncel (2021) | A three arm RCT involving 185 parents testing education against education and text messaging and control. The “I am protecting my child from the sun” plus text messaging was effective at changing parents’ sun protection behaviour | Turkey | None | No | Akdeniz University Scientific Research Projects Coordination Unit | Job titles of authors not reported | Not reported | Not reported | Not reported | Not reported | Not reported |
| 72 | Sun et al (2021) | RCT evaluating the efficacy of narrative therapy in relieving stigma in oral cancer patients. Findings indicated that narrative therapy was a promising therapeutic intervention. | China | None | No | Not reported | Job titles of authors not reported | Not reported | Not reported | Not reported | Not reported | Not reported |
| 73 | Tamayo-Morales et al (2021) | A RCT of a behavioural intervention was effective at reducing behavioural disturbance in older adults attending day care centres | Spain | None | No | Carlos III Health Institute of the Ministry of Science and Innovation; European Development Funds; Autonomous Government of Castilla y Leon. | Job titles of authors not reported | Not reported | Not reported | Not reported | Not reported | Not reported |
| 74 | Tan et al (2021) | Two-arm RCT examining if sand play assisted children with chronic illness in emotional management. Results indicate that the intervention has promise. | China | None | No | Demonstration of Technology Innovation and Application of Chongqing Science and Technology Commission (general project of social livelihood), China. | Job titles of authors not reported | Not reported | Not reported | Not reported | Not reported | Not reported |
| 75 | Teng et al (2021) | Two arm RCT examining an individualised health intervention on people with chronic kidney disease. Results indicate that participant readiness is key to efficacy of health interventions. | Taiwan | None | No | National Science Council, Taiwan | Job titles of authors not reported | Not reported | Not reported | Not reported | Not reported | Not reported |
| 76 | Tonye-Geoffroy et al (2021) | Hypnosis for chronic pain was tested in 72 patients receiving treatment with TENS. No difference between the groups were observed | France | None | No | Paramedicale Nursing Research Hospital Program. Website in French only | Job titles of authors not reported | Not reported | Not reported | Not reported | Not reported | Not reported |
| 77 | Tseng et al (2021) | Two arm RCT examining the effects of a family-centred care model for older persons with hip fracture and cognitive impairment and their family caregivers. Findings indicate that the intervention was helpful for caregivers but did not cultivate desired outcomes for patients. | Taiwan | None | No | National Health Research Institutes, Taiwan; the Healthy Aging Research Center, Chang Gung University; Higher Education Sprout Project by the Ministry of Education (MOE) in Taiwan; Chang Gung Medical Foundation; Ministry of Science and Technology, Taiwan. | Job titles of authors not reported | Not reported | Not reported | Not reported | Not reported | Not reported |
| 78 | Unal et al (2021) | Two arm RCT evaluating the effects of vapocoolant spray administration prior to subcutaneous low molecular weight heparin injection on local ecchymosis, hematoma, and pain. Results indicated limited efficacy of the intervention. | Turkey | None | No | Own account | No | Not reported | Not reported | Not reported | Not reported | Not reported |
| 79 | Valeberg et al (2021) | RCT determining the effect on pain of a psychoeducation intervention to improvement pain management following day surgery in 220 participants | Norway | None | No | Not reported | No | Not reported | Not reported | Not reported | Not reported | Not reported |
| 80 | Vargas-Porras et al (2021) | Two arm RCT testing the efficacy of a nursing intervention based on Mercer's Becoming a Mother Theory, in supporting the process of becoming a mother. The intervention group demonstrated higher scores in functional social support, perceived maternal self‐efficacy, and mother–infant bond. | Columbia | None | Yes | Not reported | Job titles of authors not reported | Not reported | Not reported | Not reported | Not reported | Not reported |
| 81 | Wang et al (2021a) | Two arm RCT exploring the feasibility and acceptability of an evidence‐based bibliotherapy protocol. Results did not indicate a significant improvement in the intervention group. | China | None | No | Not reported | Job titles of authors not reported | Not reported | Not reported | Not reported | Not reported | Not reported |
| 82 | Wang et al (2021b) | Two arm RCT assessing the effect of Chahuang ointment, on the prevention of phlebitis in patients with peripherally inserted central catheters. Results indicate that the ointment prevented phlebitis. | China | None | No | Not reported | Job titles of authors not reported | Not reported | Not reported | Not reported | Not reported | Not reported |
| 83 | Wang et al (2021c) | Two arm RCT evaluating the efficacy of the family participatory dignity therapy programme in improving the psychological well-being and family cohesion and adaptability of patients with haematologic malignancies and their family caregivers. Findings indicate the intervention was useful. | China | None | Yes | Natural Scientific Foundation Project of Fujian Province & Science and Technology Innovation Joint Fund Project of Fujian Province | Job titles of authors not reported | Not reported | Not reported | Not reported | Not reported | Not reported |
| 84 | Wu et al (2021) | Two arm examining the effect of enhanced recovery after surgery and rapid rehabilitation concepts on the outcomes of patients with haemophilia A undergoing total knee arthroplasty. The intervention group had a shorter hospital stay post-surgery. | China | None | No | Jinan Science and Technology Bureau's Clinical Medical Technology Innovation Program | Job titles of authors not reported | Not reported | Not reported | Not reported | Not reported | Not reported |
| 85 | Wyatt et al (2021) | Three arm RCT comparing home‐based reflexology and meditative practices on severity of fatigue and other symptoms among patients with cancer and informal caregivers. Both interventions were found to be equally effective | USA | None | Yes | National Cancer Institute | Job titles of authors not reported | Not reported | Not reported | Not reported | Not reported | Not reported |
| 8**6** | Yin et al (2021) | RCT evaluating effects of motor imagery training on lower limb motor function of stroke patients. Findings indicate that MIT may be useful in conjunction with routine physical therapy. | China | None | Yes | Traditional Chinese Medicine Scientific Research Fund of Zhejiang Province; Zhejiang Provincial Medical and Health Science and Technology Plan | Job titles of authors not reported | Not reported | Not reported | Not reported | Not reported | Not reported |
| 87 | Zhang et al (2021a) | Caregivers (n=125) of patients with permanent enterostomy randomised to receive either the hospital family holistic care or TAU. The experimental intervention led to improved care ability of care givers | China | None | Yes | Dreyfus Health Founding, Fujian Provincial Health Technology Project, and National Key Clinical Specialty Discipline Construction Program of China | Job titles of authors not reported | Not reported | Not reported | Not reported | Not reported | Not reported |
| 88 | Zhang et al (2021) | Two arm RCT evaluating the advantages of immediate urinary catheter removal compared with prolonged catheterization in lung cancer lobectomy. Immediate removal of urinary catheter had fewer complications and shorter hospital stay. | China | None | No | Own account | Job titles of authors not reported | Not reported | Not reported | Not reported | Not reported | Not reported |
| 89 | Zhao et al (2021) | RCT of a mixed management intervention in 182 prenatal women improved overall and exclusive breastfeeding | China | None | Yes | University funding | No | Not reported | Not reported | Not reported | Not reported | Not reported |

PPIE = Patient and Public Involvement and Engagement, TAU = Treatment as usual, COPD = Chronic Obstructive Pulmonary Disease
