# Supplementary material for: Reporting of patient and public involvement and engagement (PPIE) in clinical trials published in nursing science journals: a descriptive study
Source: Res Involv Engagem. 2021 Dec 14;7:88. doi: 10.1186/s40900-021-00331-9 (PMC8669663; doi:10.1186/s40900-021-00331-9)
Supplement: Supplementary file 3 — Additional file 3. Data extraction table. [file 40900_2021_331_MOESM3_ESM.docx]

Included trials

1. Abanes, J. J., Ridner, S. H., Dietrich, M. S., Hiers, C., & Rhoten, B. (2021). Acupuncture for Sleep Disturbances in Post-Deployment Military Service Members: A Randomized Controlled Trial. *Clinical Nursing Research*. Scopus. https://doi.org/10.1177/10547738211030602
2. Akgün Kostak, M., Kutman, G., & Semerci, R. (2021). The effectiveness of finger puppet play in reducing fear of surgery in children undergoing elective surgery: A randomised controlled trial. *Collegian*, *28*(4), 415–421. Scopus. https://doi.org/10.1016/j.colegn.2020.10.003
3. Akhlaghi, E., Babaei, S., Mardani, A., & Eskandari, F. (2021). The Effect of the Neuman Systems Model on Anxiety in Patients Undergoing Coronary Artery Bypass Graft: A Randomized Controlled Trial. *Journal of Nursing Research*, *29*(4). Scopus. https://doi.org/10.1097/JNR.0000000000000436
4. Akin, B., Yurteri Türkmen, H., Yalnız Dilcen, H., & Sert, E. (2021). The Effect of Labor Dance on Traumatic Childbirth Perception and Comfort: A Randomized Controlled Study. *Clinical Nursing Research*. Scopus. https://doi.org/10.1177/10547738211030745
5. Al-Jubouri, M. B. A., Isam, S. R., Hussein, S. M., & Machuca-Contreras, F. (2021). Recitation of quran and music to reduce chemotherapy-induced anxiety among adult patients with cancer: A clinical trial. *Nursing Open*, *8*(4), 1606–1614. Scopus. https://doi.org/10.1002/nop2.781
6. Arazi, T., Aliasgharpour, M., Mohammadi, S., Mohammadi, N., & Kazemnejad, A. (2021). Effect of a Breathing Exercise on Respiratory Function and 6-Minute Walking Distance in Patients Under Hemodialysis: A Randomized Controlled Trial. *The Journal of Nursing Research : JNR*, *29*(2), e146. Scopus. https://doi.org/10.1097/JNR.0000000000000423
7. Arjunan, P., & Trichur, R. V. (2021). The Impact of Nurse-Led Cardiac Rehabilitation on Quality of Life and Biophysiological Parameters in Patients with Heart Failure: A Randomized Clinical Trial. *Journal of Nursing Research*, *29*(1). Scopus. https://doi.org/10.1097/JNR.0000000000000407
8. Barbour, T., O’Keefe, S., & Mace, S. E. (2021). Topical Refrigerant Spray for IVs: Patient/Provider Responses - Prospective, Double-blind, Randomized Study. *Western Journal of Nursing Research*, *43*(8), 762–769. Scopus. https://doi.org/10.1177/0193945920976061
9. Basak, T., Demirtas, A., Yorubulut, S.M. Virtual reality and distraction cards to reduce pain during intramuscular benzathine penicillin injection procedure in adults: A randomized controlled trial (2021) Journal of Advanced Nursing, 77 (5), pp. 2511-2518.
10. Bielderman, A., Nieuwenhuis, A., Hazelhof, T.J.G.M., van Gaal, B.G.I., Schoonhoven, L., Akkermans, R.P., Spijker, A., Koopmans, R.T.C.M., Gerritsen, D.L. Effects on staff outcomes and process evaluation of the educating nursing staff effectively (TENSE) program for managing challenging behavior in nursing home residents with dementia: A cluster-randomized controlled trial (2021) International Journal of Nursing Studies, 120, art. no. 103982, .
11. Bozdogan Yesilot, S., Ciftci, H., & Ozcelik, Z. (2021). The effect of virtual reality on mothers’ anxiety during children’s circumcision: A randomized controlled study. *International Journal of Nursing Practice*, *27*(4). Scopus. https://doi.org/10.1111/ijn.12906
12. Bunsanong, T., Chaimongkol, N. A self-management support intervention on knee functional status and health-related quality of life among middle-age women: A randomized controlled trial (2021) Journal of Advanced Nursing, 77 (1), pp. 376-386.
13. Calvo, E., Izquierdo, S., Castillo, R., César, E., Domene, G., Gómez, A.B., Guerrero, C., Andreu-Periz, L., Gómez-Hospital, J.A., Ariza-Solé, A. Can an individualized adherence education program delivered by nurses improve therapeutic adherence in elderly people with acute myocardial infarction?: A randomized controlled study (2021) International Journal of Nursing Studies, 120, art. no. 103975,
14. Çankaya, S., & Şimşek, B. (2021). Effects of Antenatal Education on Fear of Birth, Depression, Anxiety, Childbirth Self-Efficacy, and Mode of Delivery in Primiparous Pregnant Women: A Prospective Randomized Controlled Study. *Clinical Nursing Research*, *30*(6), 818–829. Scopus. https://doi.org/10.1177/1054773820916984.
15. Cayir, E., Cunningham, T., Ackard, R., Haizlip, J., Logan, J., & Yan, G. (2021). The Effects of the Medical Pause on Physiological Stress Markers among Health Care Providers: A Pilot Randomized Controlled Trial. *Western Journal of Nursing Research*. Scopus. https://doi.org/10.1177/01939459211027657
16. Chan, H.-Y., Chang, H.-C., Huang, T.-W. Virtual reality teaching in chemotherapy administration: Randomised controlled trial (2021) Journal of Clinical Nursing, 30 (13-14), pp. 1874-1883.
17. Chen, S., Lv, C., Wu, J., Zhou, C., Shui, X., & Wang, Y. (2021). Effectiveness of a home-based exercise program among patients with lower limb spasticity post-stroke: A randomized controlled trial. *Asian Nursing Research*, *15*(1), 1–7. Scopus. https://doi.org/10.1016/j.anr.2020.08.007
18. Chew, H.S.J., Sim, K.L.D., Choi, K.C., Chair, S.Y. Effectiveness of a nurse-led temporal self-regulation theory-based program on heart failure self-care: A randomized controlled trial (2021) International journal of nursing studies, 115, p. 103872.
19. Choi, Y.-H., & Kim, S. R. (2021). The effect of uncoated paper application on skin moisture, risk of pressure injury and incidence of pressure injury in neurologic intensive care unit patients: A randomized controlled trial. *International Journal of Nursing Practice*, *27*(4). Scopus. https://doi.org/10.1111/ijn.12919
20. Chung, J. O. K., Li, W. H. C., Ho, K. Y., Lam, K. K. W., Cheung, A. T., Ho, L. L. K., Lin, J. J., & Lopez, V. (2021). Adventure-based training to enhance resilience and reduce depressive symptoms among juveniles: A randomized controlled trial. *Research in Nursing and Health*, *44*(3), 438–448. Scopus. https://doi.org/10.1002/nur.22127
21. Deng, P., Hao, L., Deng, Y., Yao, R., & Cao, Y. (2021). Pre-emptive remifentanil alleviates pain associated with tracheal suctioning in patients under mechanical ventilation and goal-directed sedation: A randomized controlled feasibility trial. *International Journal of Nursing Practice*. Scopus. https://doi.org/10.1111/ijn.12915
22. de Pinho, L.M.G., Sequeira, C.A.D.C., Sampaio, F.M.C., Rocha, N.B., Ozaslan, Z., Ferre-Grau, C. Assessing the efficacy and feasibility of providing metacognitive training for patients with schizophrenia by mental health nurses: A randomized controlled trial (2021) Journal of Advanced Nursing, 77 (2), pp. 999-1012.
23. Dincer, B., Yildirim, D. The effect of vibration stimulation on intramuscular injection pain and patient satisfaction: Single–blind, randomised controlled study (2021) Journal of Clinical Nursing, .
24. Diniz, T. S. R., Fusco, S. D. F. B., Oliveira, M. E. C. D., Nunes, H. R. D. C., & Avila, M. A. G. D. (2021). Telephonic Nurse Guidance for Colonoscopy: A Clinical Trial. *Clinical Nursing Research*, *30*(6), 762–770. Scopus. https://doi.org/10.1177/1054773821995015
25. Doğan, U., Ovayolu, N. The effect of training on treatment adherence in coronary artery patients: A single-blind randomised controlled trial (2021) Journal of Clinical Nursing, .
26. Durmuş İskender, M., & Çalışkan, N. (2021). Effect of Acupressure and Abdominal Massage on Constipation in Patients with Total Knee Arthroplasty: A Randomized Controlled Study. *Clinical Nursing Research*. Scopus. https://doi.org/10.1177/10547738211033917
27. Eslami, E., Mohammad Alizadeh Charandabi, S., Farshbaf Khalili, A., Asghari Jafarabadi, M., & Mirghafourvand, M. (2021). The effect of a lifestyle training package on physical activity and nutritional status in obese and overweight pregnant women: A randomized controlled clinical trial. *International Journal of Nursing Practice*. Scopus. https://doi.org/10.1111/ijn.12992
28. Evans, C.J., Bone, A.E., Yi, D., Gao, W., Morgan, M., Taherzadeh, S., Maddocks, M., Wright, J., Lindsay, F., Bruni, C., Harding, R., Sleeman, K.E., Gomes, B., Higginson, I.J. Community-based short-term integrated palliative and supportive care reduces symptom distress for older people with chronic noncancer conditions compared with usual care: A randomised controlled single-blind mixed method trial (2021) *International Journal of Nursing Studies,* 120, art. no. 103978, .
29. Franco-Antonio, C., Santano-Mogena, E., Sánchez-García, P., Chimento-Díaz, S., & Cordovilla-Guardia, S. (2021). Effect of a brief motivational intervention in the immediate postpartum period on breastfeeding self-efficacy: Randomized controlled trial. *Research in Nursing and Health*, *44*(2), 295–307. Scopus. https://doi.org/10.1002/nur.22115
30. Hajialibeigloo, R., Mazlum, S. R., Mohajer, S., & Morisky, D. E. (2021). Effect of self-administration of medication programme on cardiovascular inpatients’ medication adherence and nurses’ satisfaction: A randomized clinical trial. *Nursing Open*, *8*(4), 1947–1957. Scopus. https://doi.org/10.1002/nop2.870
31. Hassan, E.A., Baraka, A.A.E. The effect of reverse Trendelenburg position versus semi-recumbent position on respiratory parameters of obese critically ill patients: A randomised controlled trial (2021) Journal of Clinical Nursing, 30 (7-8), pp. 995-1002.
32. Ho, M.-H., Yu, L.-F., Lin, P.-H., Chang, H.-C., Traynor, V., Huang, W.-C., Montayre, J., Chen, K.-H. Effects of a simulation-based education programme on delirium care for critical care nurses: A randomized controlled trial(2021) Journal of Advanced Nursing, 77 (8), pp. 3483-3493.
33. Hong, P.-C., Chen, K.-J., Chang, Y.-C., Cheng, S.-M., & Chiang, H.-H. (2021). Effectiveness of Theory-Based Health Information Technology Interventions on Coronary Artery Disease Self-Management Behavior: A Clinical Randomized Waitlist-Controlled Trial. *Journal of Nursing Scholarship*, *53*(4), 418–427. https://doi.org/10.1111/jnu.12661
34. Huang, X.L., Tsao, Y., Chung, H.-C., Creedy, D.K. Effects of a mobile phone application for graduate nurses to improve central venous catheter care: A randomized controlled trial (2021) Journal of Advanced Nursing, 77 (5), pp. 2328-2339.
35. Hung, H.-M., Chiang, H.-C., & Wang, H.-L. (2021). The Impact of Gender on the Effectiveness of an Auricular Acupressure Intervention Administered to Community-Dwelling Poor Sleepers: A Cluster Randomized Controlled Trial. *Journal of Nursing Research*, *29*(3). Scopus. https://doi.org/10.1097/JNR.0000000000000427
36. Jiang, Y., Koh, K.W.L., Ramachandran, H.J., Nguyen, H.D., Lim, D.S., Tay, Y.K., Shorey, S., Wang, W. The effectiveness of a nurse-led home-based heart failure self-management programme (the HOM-HEMP) for patients with chronic heart failure: A three-arm stratified randomized controlled trial (2021) International Journal of Nursing Studies, 122, art. no. 104026, .
37. Kaplan Serin, E., & Citlik Saritas, S. (2021). The Effect of the Transtheoretical Model Based Walking Exercise Training and Follow-Up on Improving Exercise Behavior and Metabolic Control in Patients with Type 2 Diabetes. *Clinical Nursing Research*, *30*(3), 273–284. Scopus. https://doi.org/10.1177/1054773820920487
38. Kes, D., & Polat, U. (2021). The effect of nurse-led telephone support on adherence to blood pressure control and drug treatment in individuals with primary hypertension: A randomized controlled study. *International Journal of Nursing Practice*. Scopus. https://doi.org/10.1111/ijn.12995
39. Kim, Y., Lee, H., & Seo, J. M. (2021). Integrated Diabetes Self-Management Program Using Smartphone Application: A Randomized Controlled Trial. *Western Journal of Nursing Research*. Scopus. https://doi.org/10.1177/0193945921994912
40. Köse, S., & Yıldız, S. (2021). Motivational support programme to enhance health and well-being and promote weight loss in overweight and obese adolescents: A randomized controlled trial in Turkey. *International Journal of Nursing Practice*, *27*(1). Scopus. https://doi.org/10.1111/ijn.12878
41. Koundal, H., Dhandapani, M., Thakur, P., Dutta, P., Walia, R., Sahoo, S.K., Chhabra, R., Dhandapani, S. Effectiveness of dietary diabetes insipidus bundle on the severity of postoperative fluid imbalance in pituitary region tumours: A randomized controlled trial (2021) Journal of Advanced Nursing, .
42. Lee, M.-C., Wu, S.-F.V., Lu, K.-C., Wang, W.-H., Chen, Y.-Y., Chen, H.-M. Effect of patient-centred self-management programme on mental health, self-efficacy and self-management of patients with hypertensive nephropathy: A randomised controlled trial (2021) Journal of Clinical Nursing, .
43. Lee, M.-R., & Cha, C. (2021). A Mobile Healing Program Using Virtual Reality for Sexual Violence Survivors: A Randomized Controlled Pilot Study. *Worldviews on Evidence-Based Nursing*, *18*(1), 50–59. Scopus. https://doi.org/10.1111/wvn.12478
44. Lescop, K., Joret, I., Delbos, P., Briend-Godet, V., Blanchi, S., Brechet, C., Galivel-Voisine, A., Coudol, S., Volteau, C., Riche, V.-P., Cartron, E. The effectiveness of the BuzzyⓇ device to reduce or prevent pain in children undergoing needle-related procedures: The results from a prospective, open-label, randomised, non-inferiority study (2021) International Journal of Nursing Studies, 113, art. no. 103803.
45. Liao, J., LIU, G., Xie, N., Wang, S., Wu, T., Lin, Y., Hu, R., He, H.-G. Mothers’ voices and white noise on premature infants’ physiological reactions in a neonatal intensive care unit: A multi-arm randomized controlled trial (2021a) International Journal of Nursing Studies, 119, art. no. 103934, .
46. Liao, Y.-C., Hsu, L.-F., Hsieh, L.-Y., Luo, Y.-Y. Effectiveness of green tea mouthwash for improving oral health status in oral cancer patients: A single-blind randomized controlled trial (2021b) International Journal of Nursing Studies, 121, art. no. 103985,.
47. Liebergall-Wischnitzer, M., Shaphir, A., Solnica, A., Hochner-Celnikier, D. Are Paula method exercises effective for gastrointestinal reactivation post-elective cesarean delivery? Randomized controlled trial (2021) Journal of Advanced Nursing, 77 (4), pp. 2026-2032.
48. Lin, P.-J., Fanjiang, Y.-Y., Wang, J.-K., Lu, C.-W., Lin, K.-C., Cheong, I.-M., Pan, K.-Y., Chen, C.-W. Long-term effectiveness of an mHealth-tailored physical activity intervention in youth with congenital heart disease: A randomized controlled trial (2021) Journal of Advanced Nursing, 77 (8), pp. 3494-3506.
49. Liu, J., Chen, C., Liu, M., & Zhuang, S. (2021). Effects of Aerobic Exercise on Cognitive Function in Women with Methamphetamine Dependence in a Detoxification Program in Tianjin, China: A Randomized Controlled Trial. *Journal of Nursing Research*, *29*(4). Scopus. https://doi.org/10.1097/JNR.0000000000000440
50. Lyu, Q.-Y., Huang, J.-W., Li, Y.-X., Chen, Q.-L., Yu, X.-X., Wang, J.-L., Yang, Q.-H. Effects of a nurse led web-based transitional care program on the glycemic control and quality of life post hospital discharge in patients with type 2 diabetes: A randomized controlled trial (2021) International Journal of Nursing Studies, 119, art. no. 103929,.
51. Manzato, R.D.O., Ciol, M.A., Bolela, F., Dessotte, C.A.M., Rossi, L.A., Dantas, R.A.S. The effect of reinforcing an educational programme using telephone follow-up on health-related quality of life of individuals using warfarin: A randomised controlled trial (2021) Journal of Clinical Nursing, .
52. Mardani, A., Pedram Razi, S., Mazaheri, R., Haghani, S., & Vaismoradi, M. (2021). Effect of the exercise programme on the quality of life of prostate cancer survivors: A randomized controlled trial. *International Journal of Nursing Practice*, *27*(2). Scopus. https://doi.org/10.1111/ijn.12883
53. Milazi, M., Douglas, C., Bonner, A. A bundled phosphate control intervention (4Ds) for adults with end-stage kidney disease receiving haemodialysis: A cluster randomized controlled trial (2021) Journal of Advanced Nursing, 77 (3), pp. 1345-1356.
54. Morales-Fernández, Á., Jimenez-Martín, J.M., Morales-Asencio, J.M., Vergara-Romero, M., Mora-Bandera, A.M., Aranda-Gallardo, M., Canca-Sanchez, J.C. Impact of a nurse-led intervention on quality of life in patients with chronic non-malignant pain: An open randomized controlled trial (2021) Journal of Advanced Nursing, 77 (1), pp. 255-265.
55. Nesset, M.B., Bjørngaard, J.H., Whittington, R., Palmstierna, T. Does cognitive behavioural therapy or mindfulness-based therapy improve mental health and emotion regulation among men who perpetrate intimate partner violence? A randomised controlled trial (2021) International Journal of Nursing Studies, 113, art. no. 103795.
56. Oh, Y. H., & Hwang, S. Y. (2021). Individualized education focusing on self-management improved the knowledge and self-management behaviour of elderly people with atrial fibrillation: A randomized controlled trial. *International Journal of Nursing Practice*, *27*(4). Scopus. https://doi.org/10.1111/ijn.12902
57. Ok, E., & Kutlu, Y. (2021). The Effect of Motivational Interviewing on Adherence to Treatment and Quality of Life in Chronic Hemodialysis Patients: A Randomized Controlled Trial. *Clinical Nursing Research*, *30*(3), 322–333. Scopus. https://doi.org/10.1177/1054773820974158
58. Østergaard, B., Mahrer-Imhof, R., Shamali, M., Nørgaard, B., Jeune, B., Pedersen, K.S., Lauridsen, J.Effect of family nursing therapeutic conversations on patients with heart failure and their family members: Secondary outcomes of a randomised multicentre trial(2021) Journal of Clinical Nursing, 30 (5-6), pp. 742-756.
59. Pazarcikci, F., Efe, E. Effect of care programme based on Comfort Theory on reducing parental anxiety in the paediatric day surgery: Randomised controlled trial (2021) Journal of Clinical Nursing, .
60. Riera-Sampol, A., Bennasar-Veny, M., Tauler, P., Aguilo, A. Effectiveness of physical activity prescription by primary care nurses using health assets: A randomized controlled trial (2021) Journal of Advanced Nursing, 77 (3), pp. 1518-1532.
61. Sahebkar, M., Khosrojerdi, A., Rad, M., Stewart, J.J., Rastaghi, S., Assarroudi, A. Evaluation of the effect of selecting gluteal injection site on the pain injection based on anthropometric indices and body shape pattern: A randomised controlled trial study (2021) Journal of Clinical Nursing.
62. Samami, E., Elyasi, F., Mousavinasab, S. N., Shojaee, L., Zaboli, E., & Shahhosseini, Z. (2021). The effect of a supportive program on coping strategies and stress in women diagnosed with breast cancer: A randomized controlled clinical trial. *Nursing Open*, *8*(3), 1157–1167. Scopus. https://doi.org/10.1002/nop2.728
63. Shariati, E., Dadgari, A., Talebi, S. S., Mahmoodi Shan, G. R., & Ebrahimi, H. (2021). The Effect of the Web-Based Communication between a Nurse and a Family Member on the Perceived Stress of the Family Member of Patients with Suspected or Confirmed COVID-19: A Parallel Randomized Clinical Trial. *Clinical Nursing Research*. Scopus. https://doi.org/10.1177/10547738211017688
64. Shen, M.D., Li, Y.W., Xu, L.Q., Shi, H.Y., Ni, Y.Y., Lin, H.J., Li, F. Role of active cycle of breathing technique for patients with chronic obstructive pulmonary disease: A pragmatic, randomized clinical trial (2021) International Journal of Nursing Studies, 117, art. no. 103880.
65. Shin, J., Kim, G. S., & Sim, H. (2021). Comparing the effectiveness of three pain relief methods for inserting a needle into the implanted venous access chemoport: A randomized controlled trial. *International Journal of Nursing Practice*. Scopus. https://doi.org/10.1111/ijn.12974
66. Siebmanns, S., Johansson, P., Ulander, M., Johansson, L., Andersson, G., & Broström, A. (2021). The effect of nurse-led Internet-based cognitive behavioural therapy for insomnia on patients with cardiovascular disease: A randomized controlled trial with 6-month follow-up. *Nursing Open*, *8*(4), 1755–1768. Scopus. https://doi.org/10.1002/nop2.817
67. Silva, S. R. D., Reichembach, M. T., Pontes, L., Souza, G. P. E. S. C. M., & Kusma, S. (2021). Heparin solution in the prevention of occlusions in Hickman® catheters a randomized clinical trial. *Revista Latino-Americana de Enfermagem*, *29*, e3385. Scopus. https://doi.org/10.1590/1518-8345.3310.3385
68. Silva, L. R., Vasconcelos, C. T. M., Nicolau, A. I. D. O., Teles, L. M. R., Ribeiro, G. L., & Damasceno, A. K. D. C. (2021). The effect of educational technology use to guide parturient women’s companions: A randomized controlled study. *Revista Da Escola de Enfermagem*, *55*, 1–8. Scopus. https://doi.org/10.1590/S1980-220X2019022903666
69. Simonetti, V., Comparcini, D., Tomietto, M., Pavone, D., Flacco, M.E., Cicolini, G. Effectiveness of a family nurse-led programme on accuracy of blood pressure self-measurement: A randomised controlled trial (2021) Journal of Clinical Nursing.
70. Simón-López, L.C., Luquero-Bueno, S., Ovejero-Benito, M.C., Cuesta-Lozano, D., Goodman-Casanova, J.M., Vargas-Castrillón, E., Mazarro, D.O., De Miguel-Cáceres, A., Posada-Moreno, P., Zaragoza-García, I., Ortuño-Soriano, I. Benefits of the application of heat and pressure on peripheral venous cannulation in adults: A randomized controlled trial(2021) Journal of Advanced Nursing, 77 (3), pp. 1533-1545.
71. Sümen, A., Öncel, S. The effect of ‘I am Protecting my Child from the Sun’ programme on parental sun protection behaviours: Randomized controlled trial (2021) Journal of Advanced Nursing, 77 (1), pp. 387-400.
72. Sun, L., Liu, X., Weng, X., Deng, H., Li, Q., Liu, J., & Luan, X. (2021). Narrative therapy to relieve stigma in oral cancer patients: A randomized controlled trial. *International Journal of Nursing Practice*. Scopus. https://doi.org/10.1111/ijn.12926
73. Tamayo-Morales, O., Patino-Alonso, M.C., Losada, A., Mora-Simón, S., Unzueta-Arce, J., González-Sánchez, S., Gómez-Marcos, M.A., García-Ortiz, L., Rodríguez-Sánchez, E. Behavioural intervention to reduce disruptive behaviours in adult day care centres users: A randomizsed clinical trial (PROCENDIAS study) (2021) Journal of Advanced Nursing, 77 (2), pp. 987-998.
74. Tan, J., Yin, H., Meng, T., & Guo, X. (2021). Effects of sandplay therapy in reducing emotional and behavioural problems in school-age children with chronic diseases: A randomized controlled trial. *Nursing Open*. Scopus. https://doi.org/10.1002/nop2.1022
75. Teng, H.-L., Yen, M., Fetzer, S., Sung, J.-M., & Hung, S.-Y. (2021). Tailoring Health-promoting Programs for Patients with Chronic Kidney Disease: Randomized Controlled Trial. *Western Journal of Nursing Research*, *43*(2), 138–150. Scopus. https://doi.org/10.1177/0193945920942487
76. Tonye-Geoffroy, L., Mauboussin Carlos, S., Tuffet, S., Fromentin, H., Berard, L., Leblanc, J., Laroche, F. Efficacy of a combination of hypnosis and transcutaneous electrical nerve stimulation for chronic non-cancer pain: A randomized controlled trial (2021) Journal of Advanced Nursing,.
77. Tseng, M.-Y., Yang, C.-T., Liang, J., Huang, H.-L., Kuo, L.-M., Wu, C.-C., Cheng, H.-S., Chen, C.-Y., Hsu, Y.-H., Lee, P.-C., Shyu, Y.-I.L. A family care model for older persons with hip-fracture and cognitive impairment: A randomized controlled trial (2021) International Journal of Nursing Studies, 120, art. no. 103995, .
78. Unal, N., Tosun, B., Aslan, O., & Tunay, S. (2021). Effects of Vapocoolant Spray Prior to SC LMWH Injection: An Experimental Study. *Clinical Nursing Research*, *30*(2), 127–134. Scopus. https://doi.org/10.1177/1054773818825486
79. Valeberg, B.T., Dihle, A., Småstuen, M.C., Endresen, A.O., Rustøen, T. The effects of a psycho-educational intervention to improve pain management after day surgery: A randomised clinical trial (2021) Journal of Clinical Nursing, 30 (7-8), pp. 1132-1143.
80. Vargas-Porras, C., Roa-Díaz, Z. M., Hernández-Hincapié, H. G., Ferré-Grau, C., & de Molina-Fernández, M. I. (2021). Efficacy of a multimodal nursing intervention strategy in the process of becoming a mother: A randomized controlled trial. *Research in Nursing and Health*, *44*(3), 424–437. Scopus. https://doi.org/10.1002/nur.22123
81. Wang, S., Cheung, D. S. K., Leung, A. Y. M., & Davidson, P. M. (2021a). Bibliotherapy for improving caregiving appraisal of informal caregivers of people with dementia: A pilot randomized controlled trial. *Research in Nursing and Health*, *44*(4), 692–703. Scopus. https://doi.org/10.1002/nur.22143
82. Wang, X., Lv, X., Zhang, J., & Wang, Y. (2021b). Effect of Chahuang ointment on prevention of phlebitis from peripherally inserted central catheter: Randomized clinical trial. *Revista Da Escola de Enfermagem*, *55*, 1–8. Scopus. https://doi.org/10.1590/S1980-220X2019008003680
83. Wang, C., Chen, J., Wang, Y., Xu, W., Xie, M., Wu, Y., Hu, R. Effects of family participatory dignity therapy on the psychological well-being and family function of patients with haematologic malignancies and their family caregivers: A randomised controlled trial (2021c) International Journal of Nursing Studies, 118, art. no. 103922,.
84. Wu, Y., Xue, H., Zhang, W., Wu, Y., Yang, Y., & Ji, H. (2021). Application of enhanced recovery after surgery in total knee arthroplasty in patients with haemophilia A: A pilot study. *Nursing Open*, *8*(1), 80–86. Scopus. https://doi.org/10.1002/nop2.605
85. Wyatt, G., Lehto, R., Guha-Niyogi, P., Brewer, S., Victorson, D., Pace, T., Badger, T., & Sikorskii, A. (2021). Reflexology and meditative practices for symptom management among people with cancer: Results from a sequential multiple assignment randomized trial. *Research in Nursing and Health*. Scopus. https://doi.org/10.1002/nur.22169
86. Yin, X.-J., Wang, Y.-J., Ding, X.-D., & Shi, T.-M. (2021). Effects of motor imagery training on lower limb motor function of patients with chronic stroke: A pilot single-blind randomized controlled trial. *International Journal of Nursing Practice*. Scopus. https://doi.org/10.1111/ijn.12933
87. Zhang, L., Yang, X., Tian, Y., Yu, Q., Xu, Y., Zhou, D., Wu, Z., & Zhao, X. (2021a). The feasibility and advantages of immediate removal of urinary catheter after lobectomy: A prospective randomized trial. *Nursing Open*. Scopus. https://doi.org/10.1002/nop2.1006
88. Zhang, X., Lin, J.L., Gao, R., Chen, N., Huang, G.F., Wang, L., Gao, H., Zhuo, H.Z., Chen, L.Q., Chen, X.H., Li, H. Application of the hospital-family holistic care model in caregivers of patients with permanent enterostomy: A randomized controlled trial (2021b) Journal of Advanced Nursing, 77 (4), pp. 2033-2049.
89. Zhao, Y., Lin, Q., Wang, J. An evaluation of a prenatal individualised mixed management intervention addressing breastfeeding outcomes and postpartum depression: A ramdomised controlled trial (2021) Journal of Clinical Nursing, .
